# Gaze and Arrows: Does the Gaze-Following Patch in the Posterior Temporal Cortex Differentiate Social and Symbolic Spatial Cues?

**DOI:** 10.1523/ENEURO.0065-24.2024

**Published:** 2024-07-19

**Authors:** Marius Görner, Hamidreza Ramezanpour, Peter Dicke, Peter Thier

**Affiliations:** ^1^Cognitive Neurology Laboratory, Hertie Institute for Clinical Brain Research, 72076 Tübingen, Germany; ^2^GTC of Neuroscience, 72076 Tübingen, Germany; ^3^IMPRS for Cognitive and Systems Neuroscience, 72076 Tübingen, Germany; ^4^Centre for Vision Research, York University, Toronto, Ontario M3J 1P3, Canada; ^5^Werner Reichardt Centre for Integrative Neuroscience, 72076 Tübingen, Germany

**Keywords:** fMRI, joint attention, social cognition, spatial cueing

## Abstract

The gaze-following patch (GFP) is located in the posterior temporal cortex and has been described as a cortical module dedicated to processing other people's gaze-direction in a domain-specific manner. Thus, it appears to be the neural correlate of Baron-Cohen's eye direction detector (EDD) which is one of the core modules in his mindreading system—a neurocognitive model for the theory of mind concept. Inspired by Jerry Fodor's ideas on the modularity of the mind, Baron-Cohen proposed that, among other things, the individual modules are domain specific. In the case of the EDD, this means that it exclusively processes eye-like stimuli to extract gaze-direction and that other stimuli, which may carry directional information as well, are processed elsewhere. If the GFP is indeed EDD's neural correlate, it must meet this expectation. To test this, we compared the GFP's BOLD activity during gaze-direction following with the activity during arrow-direction following in the present human fMRI study. Contrary to the expectation based on the assumption of domain specificity, we did not find a differentiation between gaze- and arrow-direction following. In fact, we were not able to reproduce the GFP as presented in the previous studies. A possible explanation is that in the present study—unlike the previous work—the gaze stimuli did not contain an obvious change of direction that represented a visual motion. Hence, the critical stimulus component responsible for the identification of the GFP in the previous experiments might have been visual motion.

## Significance Statement

This study presents evidence against the notion of domain specificity of an area in the posterior temporal cortex [the gaze-following patch (GFP)] previously described to specifically serve eye gaze following. This conclusion is suggested by the finding that using arrows to identify a target object among distractors is accompanied by a comparable or even larger BOLD response than when the participants are asked to use the gaze-direction of a demonstrator’s face for target selection. The fact that even the best candidate to date, the posterior temporal GFP, does not stand up to critical scrutiny casts doubt on the assumption that the brain uses a specific module to enable gaze following, as proposed by Simon Baron-Cohen.

## Introduction

The gaze-following patch (GFP) is a circumscribed region in the posterior part of the temporal cortex which was discovered in healthy human subjects that participated in fMRI experiments in which the task was to use the gaze-direction of a demonstrator to identify a target object among distractors (Materna et al., 2008a,b; Laube et al., 2011; Marquardt et al., 2017; Kraemer et al., 2020). In contrast with the respective control condition, iris-color mapping in which the observer had to shift gaze to an object whose color corresponded to the color of the demonstrator's iris, the gaze-following condition yielded a significantly larger BOLD response within the GFP. This preference for gaze-direction suggests that the GFP might be the neural realization of Baron-Cohen's eye direction detector (EDD; Baron-Cohen, 1994). Being an integral component of his mind-reading model, Baron-Cohen proposed the EDD to be domain specific (Fodor, 1983). This implies that it exclusively processes eye-like stimuli and forwards information on eye-direction to downstream modules to form a theory of mind (ToM), a concept that captures the assignment of desires, beliefs, and intentions to another person. Electrophysiological studies in nonhuman primates (NHPs) that investigated the response preferences of individual neurons in a presumably homologous brain area in the superior temporal sulcus (STS) seemed to be in line with the assumed domain specificity of the GFP, in accordance with the central tenet of the Baron-Cohen concept (Ramezanpour and Thier, 2020).

In his work, Baron-Cohen suggested that in conjunction with shape and contrast patterns, the visual motion signal inevitably yoked with the view of an eye movement plays a crucial role in the detection of eye-gaze stimuli. Hence, under the assumption that the GFP indeed corresponds to Baron-Cohen's EDD its location in a brain region known for its role in visual motion processing appears plausible. However, one may wonder to which extent the GFP is indeed selective for motion of the eyes, a selectivity to be met in order to satisfy the assumption of domain specificity. In fact a critical examination of this question is still lacking. This is why we embarked on the current fMRI study in which we compared the BOLD activity patterns resulting from contrasting gaze-direction following and iris-color mapping with an analogous contrast: arrow-direction following versus arrow-color mapping. We predicted that the GFP should remain silent in the arrow-contrast condition if it met the premise of domain specificity.

**Figure 1.**
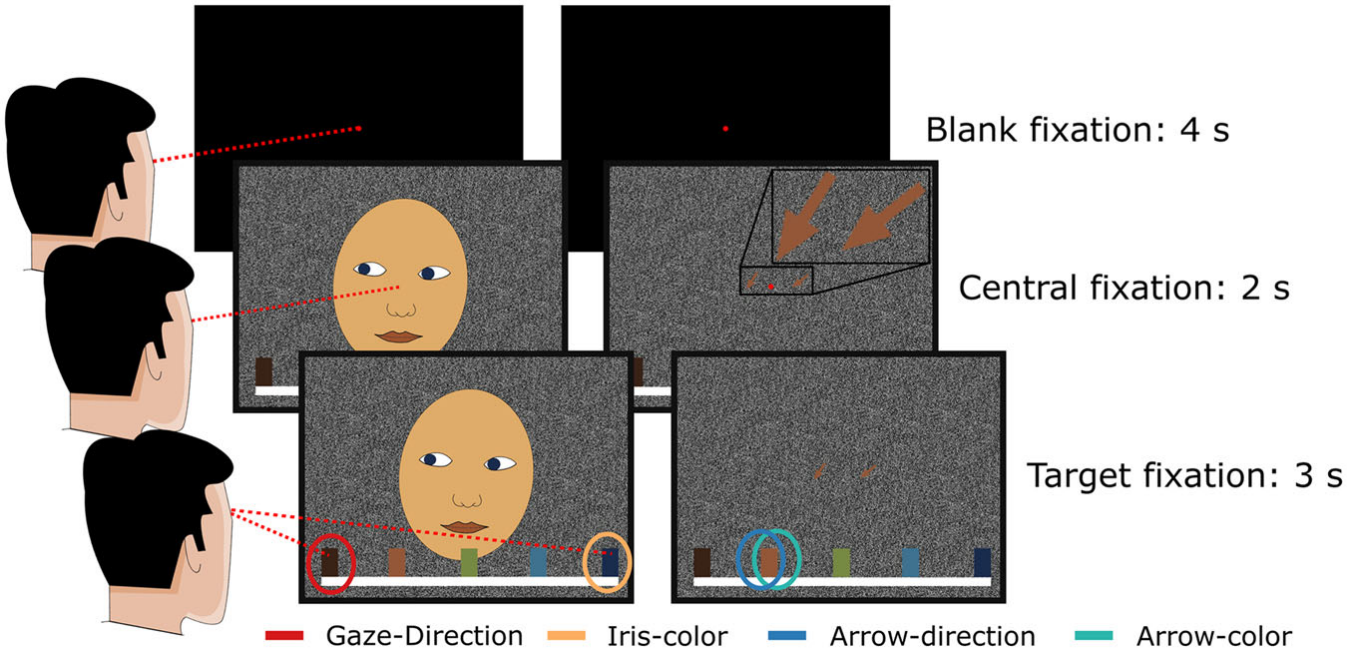
Illustration of the paradigm. The stimulus used in the experiment has been a photograph of a real person, here replaced by a drawing due to privacy reasons. The left and right columns display the sequence of events during a gaze-following/iris-color mapping and an arrow-direction/arrow-color mapping trial, respectively. Each trial started with a blank fixation period followed by the presentation of the cue. In each individual trial, the demonstrator’s face and the arrows look/point toward a randomly selected target, and the color of the iris and the arrows, chosen independently of each other, matched one of the targets. After cue presentation, the participants had to wait with their response until the central fixation dot disappeared.

In this study, we tested 20 healthy human participants using the same stimuli as in Marquardt et al. (2017) with an important modification: while individual trials in the original version of the study always started with the demonstrator's overt gaze directed toward the participant followed by a second frame depicting the demonstrator looking toward the target object, in the current study, the initial part was replaced by a blank screen. This bears the consequence that no overt gaze shift is seen as the blank screen is directly followed by the demonstrator's gaze directed toward the target (Fig. 1). Hence, the spatiotemporal discontinuity (apparent motion) of the two views of the eyes in the original version which created the impression of a saccadic gaze shift was absent. This modification was necessary to allow fully analogous sequences in the gaze and arrow conditions, since in the two-dimensional views of arrows, there is no equivalent orientation to the gaze being directed toward the participant while still being recognizable as arrows. The presence of apparent motion (Wertheimer, 1912; Ramachandran and Anstis, 1986) in the original version of the paradigm had an important consequence; by contrasting the gaze-following condition with the respective control condition—e.g., iris-color mapping—we implicitly contrasted a stimulus component that comprised a motion event (the gaze shift) with a component that comprised a color change requiring to ignore the motion event. Or, to put it differently, in the gaze-following condition, the stimulus component that was behaviorally relevant, i.e., the gaze-direction, was intrinsically linked to visual motion, while in the iris-color condition, it was not. It has been shown that both electrophysiological and BOLD signatures of visual motion in parts of the posterior STS are boosted whenever motion patterns are behaviorally relevant (Stemmann and Freiwald, 2016, 2019). This raises the question if the human GFP as described by Marquardt et al. and other studies as well as the monkey's homolog may be the result of this implicit contrast between behaviorally relevant motion and a control condition, lacking behaviorally relevant motion cues. Therefore, in the current study, the first question was whether we are able to reproduce the GFP despite the absence of any motion information provided by the stimulus. Second, we compared the activity patterns emerging from the contrast gaze-direction > iris-color with those resulting from the contrast arrow-direction > arrow-color as well as with the location of the GFP as reported by Marquardt et al. (2017). We expected that if the GFP is domain specific and does not depend on visual motion, we would not find overlapping activation between the two contrasts at the expected location of the GFP at a given statistical threshold. Moreover, for each condition, we estimated the hemodynamic response functions (HRFs) based on the GFP region of interest (ROI) reported by Marquardt et al. and based on a ROI stemming from a search for visual motion in the Neurosynth (Yarkoni, n.d.) database.

## Materials and Methods

### Participants

Twenty-three healthy participants were recruited, three of which canceled the experiment prematurely. Of the remaining 20 participants, 12 were female and 8 male and had an average age of 31.5 years with a standard deviation of 3.6 years. Each participant participated in two sessions. The first was conducted outside the scanner to familiarize the participant with the task. Participants gave written consent to the procedures of the experiment. The study was approved by the local Ethics Review Board and was conducted in accordance with the principles of human research ethics of the Declaration of Helsinki.

### Paradigm and setup

The paradigm consisted of two stimuli types with two conditions, each. One stimulus type consisted of a photo of a face and the other of two arrows that were displayed on a computer monitor. Below the stimuli, five differently colored squares were shown, which served as gaze targets. Each trial started with a black screen with a red fixation dot in the middle which was shown for 4 s. After this, either the face or the arrows were displayed, still with the red fixation dot in the center of the monitor. The face looked in the direction of one of the squares, the iris-color matched one of the colors of the squares, the arrows pointed toward one of the squares, and their color matched one of the squares as well. After 2 more seconds, the fixation dot disappeared which was the go-cue telling the participants that they now have to shift their own visual focus onto the target square defined by the current experimental condition. Conditions were organized in blocks such that the participants always had to identify the target square by the same stimulus features for 20 consecutive trials. Which stimulus feature mattered was announced by a written instruction before each block. Four blocks made up a run within which each condition occurred in randomized order. In total, four runs had to be completed, such that each condition was represented by 80 trials.

During the experiment, participants lay in the scanner and viewed the stimulus monitor via a mirror system. The distance between the participants’ eyes and the monitor was ∼190 cm, and it covered ∼20° of the field of view in the horizontal and ∼12° in the vertical. Additionally, eye-tracking data were recorded during the fMRI experiment. However as the data suffered from bad quality, they could not be analyzed consistently. In a separate session, participants were familiarized with the task outside of the scanner.

### Data collection

MR images were acquired in a 3 T scanner (Siemens Magnetom Prisma) with a 20-channel phased-array head coil. The head of the subjects was fixed inside the head coil by using plastic foam cushions to avoid head movements. An AutoAlign sequence was used to standardize the alignment of images across sessions and subjects. A high-resolution T1-weighted (T1w) anatomic scan (MP-RAGE, 176 × 256 × 256 voxel, voxel size 1 × 1 × 1 mm) and local field maps were acquired. Functional scans were conducted using a T2*-weighted echo-planar multibanded 2D sequence (multiband factor = 2; TE = 35 ms; TR = 1,500 ms; flip angle = 70°) which covers the whole brain (44 × 64 × 64 voxel, voxel size 3 × 3 × 3 mm, interleaved slice acquisition, no gap).

### Preprocessing

Results included in this manuscript were preprocessed using fMRIPrep 1.5.2 (Esteban et al., 2019, 2022).

#### Copyright waiver

The below boilerplate text was automatically generated by fMRIPrep with the express intention that users should copy and paste this text into their manuscripts unchanged. It is released under the CC0 license.

#### Anatomical data preprocessing

The T1w image was corrected for intensity nonuniformity with N4BiasFieldCorrection (Tustison et al., 2010), distributed with ANTs 2.2.0 (Avants et al., 2008), and used as T1w reference throughout the workflow. The T1w reference was then skull stripped with a Nipype implementation of the antsBrainExtraction.sh workflow (from ANTs), using OASIS30ANTs as the target template. Brain tissue segmentation of the cerebrospinal fluid (CSF), white matter (WM), and gray matter (GM) was performed on the brain-extracted T1w using fast (Zhang et al., 2001; FSL 5.0.9). Brain surfaces were reconstructed using recon-all (Dale et al., 1999; FreeSurfer 6.0.1), and the brain mask estimated previously was refined with a custom variation of the method to reconcile ANTs-derived and FreeSurfer-derived segmentations of the cortical gray matter of Mindboggle (Klein et al., 2017). Volume-based spatial normalization to one standard space (MNI152NLin2009cAsym) was performed through nonlinear registration with antsRegistration (ANTs 2.2.0), using brain-extracted versions of both the T1w reference and the T1w template. The following template was selected for spatial normalization: ICBM 152 Nonlinear Asymmetrical template version 2009c (Fonov et al., 2009; TemplateFlow ID: MNI152NLin2009cAsym).

#### Functional data preprocessing

For each of the four BOLD runs found per subject (across all tasks and sessions), the following preprocessing was performed. First, a reference volume and its skull-stripped version were generated using a custom methodology of fMRIPrep. A deformation field to correct for susceptibility distortions was estimated based on a field map that was coregistered to the BOLD reference, using a custom workflow of fMRIPrep derived from D. Greve's epidewarp.fsl script and further improvements of HCP Pipelines (Glasser et al., 2013). Based on the estimated susceptibility distortion, an unwarped BOLD reference was calculated for a more accurate coregistration with the anatomical reference. The BOLD reference was then coregistered to the T1w reference using bbregister (FreeSurfer) which implements boundary-based registration (Greve and Fischl, 2009). Coregistration was configured with six degrees of freedom. Head motion parameters with respect to the BOLD reference (transformation matrices and six corresponding rotation and translation parameters) are estimated before any spatiotemporal filtering using mcflirt (Jenkinson et al., 2002; FSL 5.0.9). BOLD runs were slice-time corrected using 3dTshift from AFNI 20160207 (Cox and Hyde, 1997). The BOLD time series were resampled to surfaces on the following spaces: fsaverage5. The BOLD time series (including slice-timing correction when applied) were resampled onto their original, native space by applying a single, composite transform to correct for head motion and susceptibility distortions. These resampled BOLD time series will be referred to as preprocessed BOLD in original space, or just preprocessed BOLD. The BOLD time series were resampled into standard space, generating a preprocessed BOLD run in [“MNI152NLin2009cAsym”] space. First, a reference volume and its skull-stripped version were generated using a custom methodology of fMRIPrep. Several confounding time series were calculated based on the preprocessed BOLD: framewise displacement (FD), derivative of root mean square variance over voxel (DVARS), and three region-wise global signals. FD and DVARS are calculated for each functional run, both using their implementations in Nipype [following the definitions by Power et al. (2014)]. The three global signals are extracted within the CSF, the WM, and the whole-brain masks. Additionally, a set of physiological regressors were extracted to allow for component-based noise correction (CompCor; Behzadi et al., 2007). Principal components are estimated after high-pass filtering the preprocessed BOLD time series (using a discrete cosine filter with 128 s cutoff) for the two CompCor variants: temporal (tCompCor) and anatomical (aCompCor). tCompCor components are then calculated from the top 5% variable voxels within a mask covering the subcortical regions. This subcortical mask is obtained by heavily eroding the brain mask, which ensures it does not include cortical GM regions. For aCompCor, components are calculated within the intersection of the aforementioned mask and the union of CSF and WM masks calculated in the T1w space, after their projection to the native space of each functional run (using the inverse BOLD-to-T1w transformation). Components are also calculated separately within the WM and CSF masks. For each CompCor decomposition, the *k* components with the largest singular values are retained, such that the retained components’ time series are sufficient to explain 50% of variance across the nuisance mask (CSF, WM, combined, or temporal). The remaining components are dropped from consideration. The head motion estimates calculated in the correction step were also placed within the corresponding confound file. The confound time series derived from head motion estimates and global signals were expanded with the inclusion of temporal derivatives and quadratic terms for each (Satterthwaite et al., 2013). Frames that exceeded a threshold of 0.5 mm FD or 1.5 standardized DVARS were annotated as motion outliers. All resamplings can be performed with a single interpolation step by composing all the pertinent transformations (i.e., head motion transform matrices, susceptibility distortion correction when available, and coregistrations to anatomical and output spaces). Gridded (volumetric) resamplings were performed using antsApplyTransforms (ANTs), configured with Lanczos interpolation to minimize the smoothing effects of other kernels (Lanczos, 1964). Nongridded (surface) resamplings were performed using mri_vol2surf (FreeSurfer).

Many internal operations of fMRIPrep use Nilearn 0.5.2 (Abraham et al., 2014), mostly within the functional processing workflow. For more details of the pipeline, see the section corresponding to workflows in fMRIPrep’s documentation.

### Analysis

#### Contrasts

For each participant, we computed a general linear model (GLM) for the combination of all runs (first-level) to obtain the respective β-images for each condition using the Python package Nilearn (Nilearn, n.d.). For modeling, we aligned the onsets of each trial to the onsets of the spatial/color cue (4 s after the actual trial onset, starting with the blank screen) and set the stimulus duration to 0, i.e., we modeled the relevant stimulus component as a single event since it is not changing over time. To mitigate the effects of motion artifacts and other noise sources, the nuisance regressors global_signal, csf, white_matter, trans_x, trans_y, trans_z, rot_x, rot_y, and rot_z and their respective first derivatives estimated by fMRIPrep were included in the design matrices. As the model for the HRF, we used the glover* + *derivative* + *dispersion provided by Nilearn and included a polynomial drift model of order 3 to remove slow drifts in the data. Further, we masked the data with the average across run's mask images provided by fMRIPrep and applied smoothing with fwhm* *= 8 mm.

For each participant, the resulting first-level β-images will be used to compute the contrasts gaze-direction*–*iris-color and arrow-direction*–*arrow-color. The resulting effect-size images (Nilearn terminology) of each contrast were fed into second-level analyses that were fitted as one-sample *t* tests. The resulting second-level contrasts were thresholded with *p* < 0.001 [false positive rate (fpr)] and *p* < 0.01 (fpr). All coordinates reported in this manuscript refer to the MNI space. For each of the reported coordinates, a Neurosynth search was conducted, and we reported the first five associations.

#### ROI definition and HRF estimation

**Figure 2.**
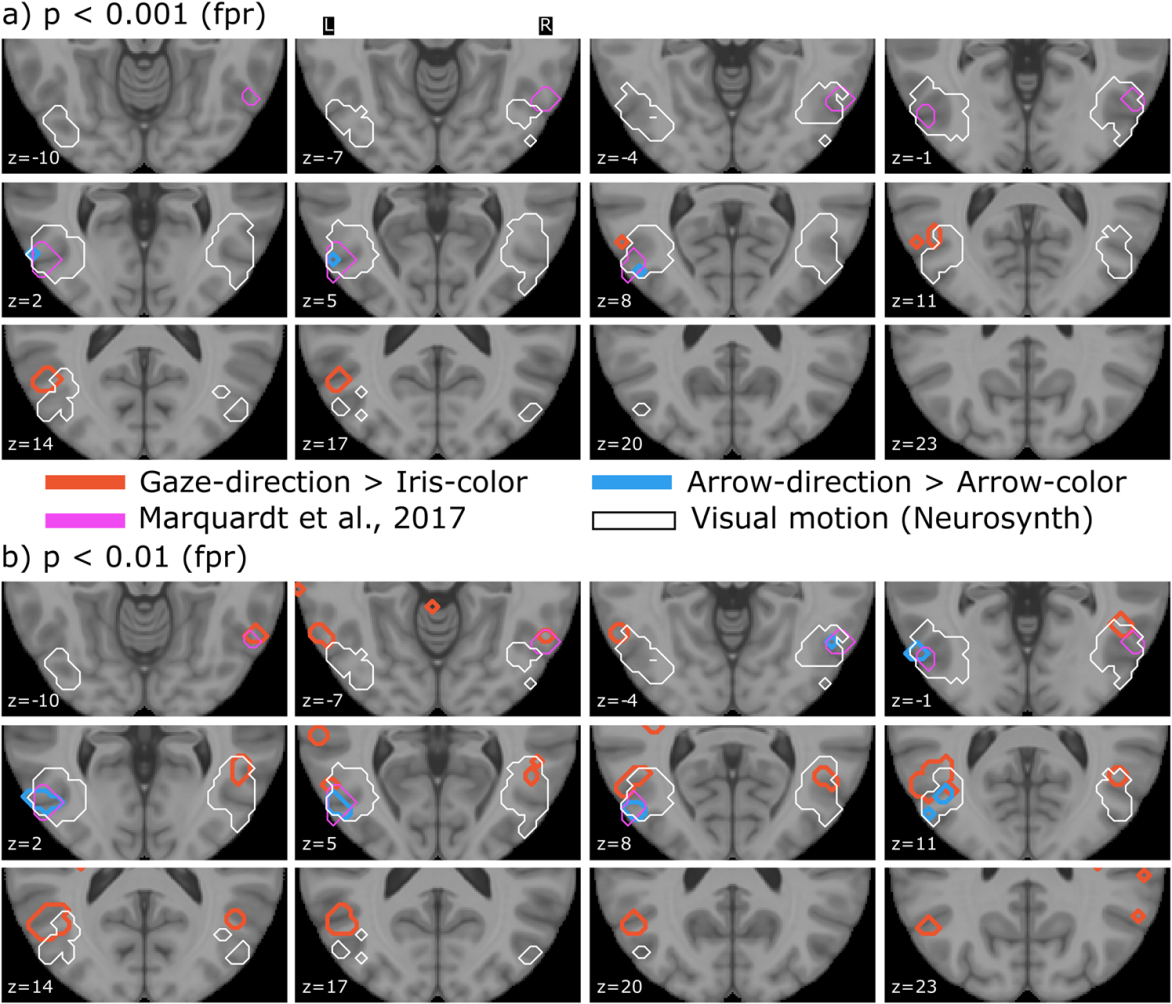
Both panels ***a*** and ***b*** show the same contrasts/ROIs at two threshold levels. White encircled is the visual motion ROI (Yarkoni, n.d.) and pink encircled is the mGFP (Marquardt et al., 2017). Blue-encircled areas belong to the arrow contrast and red-encircled areas to the gaze contrast at the respective statistical thresholds.

To define the GFP ROI, we extracted the activity patch of both hemispheres as reported by Marquardt et al. (2017) and converted them into mask images. To define a ROI corresponding to brain areas that process visual motion, we searched the Neurosynth database (term-based meta-analysis) using the term visual motion. After downloading the result (association test), we extracted the largest components in both hemispheres spanning the posterior temporal cortex and converted them into mask images. The left GFP consists of 76 voxels and the right of 61 voxels, and the visual motion ROIs consist of 377 (left) and 306 (voxels). All ROIs are plotted along with the contrasts described above in Figure 2.

For each participant and ROI, we estimated the hemodynamic response of each condition. To do so, the BOLD signals of each ROI (averaged across voxels) were extracted. Prior to signal extraction, the BOLD images were denoised using the same nuisance regressors as used to fit the GLMs described above. To recover the condition-specific hemodynamic responses, we deconvolved the signals using the nideconv package (Hollander and Knapen, 2017). We applied the Fourier basis set with nine regressors over a period of 19.5 s starting from spatial/color cue onset, again omitting the blank screen period in the beginning of each trial. Individual models were fitted using the standard settings of nideconv's fitting method. The first-level response estimates were then fed into the group-level model using nideconv's GroupResponseFitter functionality using the same settings as for the first-level. As a result, we obtain the estimated HRFs together with the 95% credible intervals (CIs) of the estimates. Periods in which the CIs do not include an activation level of 0 (au) are considered to be statistically different from 0 at the 5% level.

### Resources

Analysis code is available under https://github.com/maalaria/fMRIus/tree/gaze-arrows. The dataset is available under https://doi.org/10.18112/openneuro.ds005203.v1.0.0.

## Results

### Behavior

Because of insufficient quality of the eye tracking, we were only able to analyze the oculomotor behavior in ∼38% of all trials. The performance (∼80–90% correct responses) in this fraction of the data matched the expected performance observed in previous experiments (∼80%) using the same stimuli in all experimental conditions (Marquardt et al., 2017). Further, the eye-tracking camera enabled us to monitor participants’ behavior during the experiment, giving us confidence that participants generally complied with the task requirements. Before the fMRI session, participants underwent a practice session in which their behavior was closely monitored, and corrected, as well. This, together with the fact that the task was easy and intuitive, makes us confident that the number of error trials was indeed small and, thus, can be ignored as in previous experiments (Marquardt et al., 2017).

### Contrasts

At a threshold of *p* < 0.001 (uncorrected; Fig. 2, top) contrasting gaze-direction following with iris-color mapping (regions encircled in red in Fig. 2) did not yield activity overlapping with the GFP [as described by Marquardt et al. (2017); from now on, we refer to the GFP ROI as defined by Marquardt et al. (2017) as mGFP—the region encircled in pink in Fig. 2]. Contrasting arrow-direction following with arrow-color mapping (regions encircled in blue in Fig. 2) yielded activity in the left hemisphere that overlaps with the mGFP therein. The coordinates of the local maximum of this patch are (*x*, *y*, *z*) = (−54, −72, 6). The first five Neurosynth (Yarkoni, n.d.) associations for this location are motion, videos, v5, mt, and visual.

At this threshold, the gaze-iris contrast yielded an activity dorsal to the mGFP beginning to emerge around *z* = 8 and extending toward *z* = 18 (encircled red in Fig. 2). The local maximum of this patch is located at (*x*, *y*, *z*) = (−51, −63, 15). The first five associations for this location are action observation, intentions, mentalizing, social, and temporoparietal.

The mGFP as well as the activity related to the arrows contrast are falling within the region of the visual motion ROI (regions encircled in white in Fig. 2.) given by the Neurosynth database. Activity related to the gaze-iris contrast is slightly more frontal to this ROI.

Liberalizing the threshold to *p *< 0.01 (uncorrected; Fig. 2, bottom) additionally gave rise to patches close to or overlapping with the mGFP for the gaze-iris contrast in both hemispheres [peak coordinates: (*x*, *y*, *z*) = (−60, −60, −3) and (*x*, *y*, *z*) = (57, −60, −6)]. Neurosynth associations with these two locations are word form, judgment task, interactive, semantics, and timing (left) and visual, unfamiliar, objects, multisensory, and visually (right). Further, a patch dorsal to the mGFP emerged in the right hemisphere at this threshold for this contrast. This patch had one ventral peak at (48, −54, 0) and a dorsal peak at (48, −58, 12) which closely matched the dorsal activity in the left hemisphere at (−51, −63, 15) already visible at the more conservative threshold of *p* < 0.001. The Neurosynth associations for (48, −54, 0) are unfamiliar, motion, social interactions, gestures, and preparatory and for (48, −58, 12) motion, video clips, gaze, and action observation.

At the more liberal threshold, the activity patch at (−54, −72, 6) associated with arrow-direction following was more extended and matched the mGFP, especially in the left hemisphere. In the right hemisphere, a small activity patch emerged at this threshold at (51, −63, −3) falling into the area of the mGFP as well. The Neurosynth associations for (51, −63, −3) are visual, motion, objects, movements, and action observation.

Table 1 lists the peak coordinates as well as the Neurosynth associations of the activations found in this study as well as the GFP locations reported in two preceding studies on the GFP.

**Table 1.** List of locations of the GFP reported in earlier studies as well as locations in its vicinity found in this study

			*x*	*y*	*z*	Neurosynth associations
Materna et al. (2008a)		RH	51	−63	6	Motion, video, action observation, gestures, perception
		LH	−54	−61	7	Actions, motion, perception, audiovisual, recognize
Marquardt et al. (2017)		RH	50	−64	−3	Visual, motion, objects, action observation, viewing
		LH	−55	−68	1	Object, visual, movements, complex, action
This study	Gaze-iris	RH 0.01 (fpr) Connected	48	−58	12	Motion, video clips, gaze, action observation
			48	−54	0	Unfamiliar, motion, social interactions, gestures, preparatory
			57	−60	−6	Visual, unfamiliar, objects, multisensory, visually
		LH 0.01 (fpr)	−60	−60	−3	Word form, judgment task, interactive, semantics, timing
		LH 0.001 (fpr)	−51	−63	15	Action observation, intentions, mentalizing, social, ToM
	Arrow-color	RH 0.01 (fpr)	51	−63	−3	Visual, motion, objects, movements, action observation
			−42	−66	12	Motion, visual, visual motion, mental states, negative neutral
		LH 0.001 (fpr)	−54	−72	6	Motion, videos, visual, eye movements, viewing
			−48	−78	9	Visual, motion, gaze, action observation, videos

Coordinates are in the MNI space. For each location, we report the first five functional associations provided by neurosynth.org (Yarkoni, n.d.).

### HRF estimates

**Figure 3.**
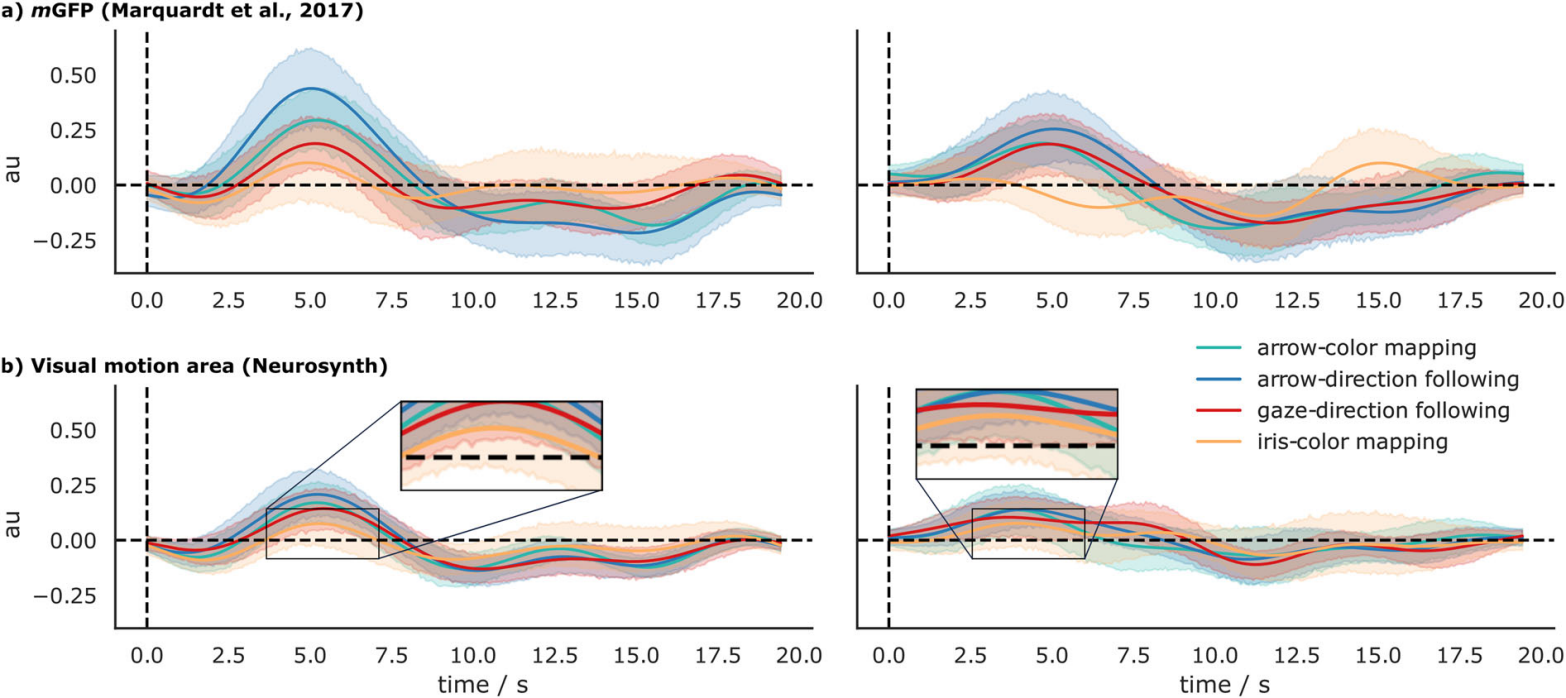
In ***a*** and ***b***, the HRF estimates for the four different conditions in the left- and right-hemispheric mGFP and visual motion ROI, respectively, are shown. Shaded areas represent the 95% CIs. Especially in the left mGFP, the dominance of activity related to the arrow-direction following condition is obvious.

Figure 3 shows the estimated HRFs and the 95% CIs for each condition for the mGFP ROI and the ROI based on a search for visual motion in the Neurosynth database. The HRFs for the mGFP show a statistically significant (95% CIs do not include the activation level of 0) deflection ∼5fs after cue onset in both hemispheres in all conditions but the iris-color condition. This matches the temporal progression of the canonical HRF which describes the expected BOLD signal in response to a given impulse stimulus (Lindquist et al., 2009). Though the difference is not statistically significant at the 5% level, the estimated activation in the arrow-direction condition is nearly twice as large as in the gaze-direction following the condition in the left hemisphere. This difference is not present in the right hemisphere.

In the visual motion ROI, the activation level is overall smaller than in the mGFP but still significant at the 5% level (Fig. 2, inlets, bottom) for all conditions but the iris-color condition in both hemispheres at ∼5 s.

## Discussion

In this study, we asked if the GFP—a brain area described in several studies as a domain-specific module dedicated to the processing of others’ gaze-direction (Materna et al., 2008a,b; Laube et al., 2011; Marquardt et al., 2017; Kraemer et al., 2020; Ramezanpour and Thier, 2020)—is indeed domain specific or if it is also active when the participants use arrows instead of the gaze of a demonstrator to identify a target object. We used the same portraits as stimuli that were used in previous experiments based upon which the GFP was originally defined but had to introduce a modification to the temporal structure of the task to allow a direct comparison between the gaze and the arrow condition. In the original version of the task that was used in human studies (Materna et al., 2008a; Marquardt et al., 2017; Kreamer et al., 2020) as well as in studies with NHP (Kamphuis et al., 2009; Ramezanpour and Thier, 2020), each trial started with a frame displaying the demonstrator looking straight ahead toward the participant, followed by a second frame displaying the demonstrator looking toward the target object. Whenever two consecutive frames feature a coherent shift of pixels that does not shatter the correspondence of the depicted scene in the two frames, the visual system interprets the sequence as motion (Wertheimer, 1912; Ramachandran and Anstis, 1986). In the current study, the initial frame was replaced by a black screen which was directly followed by the frame displaying the demonstrator looking toward the target object. In light of this modification, the first question here was whether we can replicate the GFP despite the absence of apparent motion in the form of the gaze shift. We have to answer that in the negative. Even though the sample size and the MRI scanner used in this study were the same as in previous studies, at the same statistical threshold used by Marquardt et al. (2017) and even at more liberal thresholds, we could not detect any activation for the gaze-direction minus iris-color contrast that coincided with the GFP. However, due to a lack of a positive control for the role of apparent motion, we cannot confirm that it is indeed its lack causing the failure to reproduce the GFP. We propose, however, that this lack is compensated by the second analysis, involving the estimation of HRFs, which demonstrated a significant activation in the mGFP ROI in all conditions but the iris-color mapping condition [Fig. 3; with “mGFP” we denote the GFP ROI as delineated based on the data of Marquardt et al. (2017); a more detailed discussion follows further below].

Even at the statistically most liberal threshold that we applied (*p *< 0.01, uncorrected), there was only a small patch related to the contrast gaze-direction following minus iris-color mapping partially overlapping with the mGFP (Fig. 2*b*, Table 1). Surprisingly, however, the contrast arrow-direction following minus arrow-color mapping yielded activity patches overlapping with the mGFP for both statistical thresholds (Fig. 2, Table 1). This area of relatively stronger activity during arrow-direction following compared with arrow-color mapping as well as the mGFP falls within parts of a ROI that are associated with the search term visual motion in the neurosynth.org (Yarkoni, n.d.) database (Fig. 2, white encircled areas). When searching for the functional associations with the locations activated in the arrow contrast, Neurosynth then also provides visual, motion, video, eye movements, etc., as expected (Table 1).

Activity only detectable for the gaze but not the arrow contrast at *p *< 0.001 (uncorrected) can be found dorsal to the mGFP in the left hemisphere with its center at *x*, *y*, *z* = −51, −63, 15 (MNI). Anatomically, this location corresponds to BA39 or the temporoparietal junction. Searching for functional associations of this location at neurosynth.org (Yarkoni, n.d.) yields action observation, intention, mentalizing, social, and ToM as the first five results (Table 1). This location was not reported by Marquardt et al. (2017) or Materna et al. (2008a) to be activated during gaze following but fits to the idea that perceiving the other's gaze evokes the assignment of intentions (Baron-Cohen, 1994, n.d., Perrett and Emery, 1994; Nummenmaa and Calder, 2009).

These results provide a surprising picture of the GFP. First, a gaze stimulus lacking apparent motion does not yield a relatively stronger activation when compared with the color mapping condition at the mGFP location. Second, however, arrows pointing toward the target, despite lacking apparent motion as well, yield detectable activity confined to a region very well overlapping with the mGFP when contrasted with the color mapping condition. Note, however, that the more conservative threshold of *p* < 0.001 is not corrected for multiple comparisons as well.

To capture the activity within the mGFP ROI beyond the contrasts and compare it to the activity within the visual motion ROI, we modeled the HRFs for each of the conditions and hemispheres individually (Fig. 3). We found that all conditions but the iris-color mapping condition yielded a significant activation in all ROIs. Moreover, a comparable pattern is apparent in all ROIs, with the arrow-direction condition featuring the strongest activation (Fig. 3, blue curves). However, there is no statistically significant difference between any condition as the 95% CIs overlap at all time points. Comparison of the general activity patterns obtained for both ROIs shows their similarity, which is not surprising since the mGFP overlaps nearly completely with the more lateral part of the visual motion ROI. The overall smaller amplitude of the HRF for the latter ROI is likely due to the fact that the visual motion ROI was about five times larger than the mGFP and therefore most probably comprised voxels which added task-independent signals.

This sheds new light on the negative result of the contrast analysis and raises the question of what triggers the activation of mGFP, given the absence of apparent motion. Taking the results of the contrast analysis and HRF estimates together, we can think of three possible explanations. First, it might be a nonspecific response within the visual system to the switch from a blank screen to the stimulus image. Indeed, it was shown that the BOLD activity in the early visual cortex is positively correlated with scene complexity (Groen et al., 2018). Unfortunately, it appears unclear whether the apparently higher visual complexity/size of the face stimulus compared with the arrows should lead to a stronger activation in the visual system, or whether this is to be expected from the less conspicuous arrows, which are for this very reason—corresponding to a de facto higher complexity—more difficult to recognize, thus resulting in a higher workload. On that ground, we cannot exclude this possibility. The second possibility is that the location corresponding to the mGFP ROI is relevant for the processing of the behaviorally relevant orientation of objects—independent of the type of object. However, we are not aware of any studies that would directly support this hypothesis. Some studies suggest neural tuning to object orientations in area V4 (Moore, 1999) as well as in the parietal and frontal areas (Henderson and Serences, 2019). But because their paradigms and ours are very different, it is not clear how the results can be related to each other. As a third possibility, we propose that the observed activity patterns can be attributed to an effect described as implied motion. Both psychophysical (Faivre and Koch, 2014) and fMRI studies (Kourtzi and Kanwisher, 2000; Senior et al., 2000) have demonstrated that static images of moving objects elicit experimental effects that are known from the perception of visual motion (motion adaptation). How could this effect explain our results, given the fact that the stimulus images used here do not depict moving objects? Guterstam et al. published a series of studies [Guterstam et al., 2019; Guterstam and Graziano, 2020a,b; compare a Comment Letter (Görner et al., 2020)] in which they demonstrated that viewing a static image of a diagram face looking toward an object is indeed able to evoke motion adaptation in the viewer. In an fMRI study, Guterstam et al., (2020) furthermore showed that these behavioral effects are accompanied by activity within the human MT+ complex. However, in their data, they find that these effects are unique to gaze and are not present if the stimulus is an arrow, contradicting our results that demonstrate a significant BOLD response to a face gazing at an object as well as to arrows pointing at an object. A study by Lorteije et al. (2011), however, presented evidence that the effects typically attributed to implied motion can be attributed to low-level features of the stimuli such as orientation and size. Thus, the effect we find here for arrows seems plausible, though the interpretation would change accordingly. In fact, the results from Lorteije et al. are related to the second explanation (orientation tuning) that we describe above.

Other authors have used arrows as attentional cues and compared them to gaze in spatial cueing tasks inspired by Posner (1980) before us. However, due to the differences in the tasks, no conclusive comparisons can be made with our results, yet they help to contextualize our findings. For example Hietanen et al. (2006) found that gaze and arrows, when used as central endogenous cues, elicit the same behavioral effects, but the BOLD activations differ. Activity related to arrows was much more widespread involving all cortical lobes, while activity related to gaze was confined to the inferior and middle occipital gyri, however, in some distance to the mGFP. A study by Callejas et al. (2014), on the other hand, reported that largely the same neural networks encode gaze and arrow cues but that some differential modulations occurred in parts of the intraparietal sulcus and in the MT+ region. Interestingly, they found that the MT+ region exhibited effects only in relation to arrow cues. In that both studies report a stronger BOLD activation associated with arrow cues compared with gaze cues, our results are consistent. However, other than in Callejas’ study, we also find a significant, albeit smaller, activation in the MT+ region in response to static gaze cues. As an explanation for the greater activity associated with the arrow stimuli, the authors suggest the greater automaticity of responses to gaze stimuli, as well as that the processing of arrow stimuli could be more demanding. Given that the gaze of others is ubiquitous from birth and plays a crucial role in ontogeny, the processing of faces and gaze is likely to be highly optimized resulting in smaller signal changes when investigated using fMRI. There are two neuropsychological cases that are of great interest as well. One is reported by Akiyama et al. (2006) and consists of an impairment of using gaze but not arrows as a spatial cue after a lesion to the (entire) right superior temporal gyrus. This case confirms that the superior temporal region, which was reported to be involved in face perception (Kanwisher et al., 1997; Halgren et al., 1998; Haxby et al., 1999), especially in the perception of changeable aspects of faces such as eye and mouth movements (Puce et al., 1998; Hoffman and Haxby, 2000), is an integral component and that its lack leads to impairments in tasks in which eye recognition plays a crucial role. The other case is reported in two studies by Vecera and Rizzo (2004, 2006) and points toward another brain region that may be crucial for the perception and interpretation of gaze cues as well—the frontal lobe. They report the case of a patient with frontal lobe damage who had lost the ability to direct attention to peripheral locations volitionally through endogenous cues such as words and gaze, but could still attend automatically to exogenous cues. Based on this, they suggest an association hypothesis that the gaze of another person is understood, analogous to words, through associations of gaze-directions with locations in space.

All this leaves a situation involving a number of perspectives impossible to reconcile at this point. Given the lack of a positive control for the role of visual motion, we cannot be sure that the reason for the negative finding in the contrast analysis is indeed the absence of visual motion in the stimuli. However, the HRF estimates do provide strong evidence that the GFP does not differentiate between arrow- and gaze-stimuli and if it should differentiate after all that arrow-stimuli elicit at least stronger responses than gaze-stimuli, contradicting the previous assumption of a specific functional role of the GFP in gaze following.

## Synthesis

Reviewing Editor: Niko Busch, Westfalische Wilhelms-Universitat Munster Decisions are customarily a result of the Reviewing Editor and the peer reviewers coming together and discussing their recommendations until a consensus is reached. When revisions are invited, a fact-based synthesis statement explaining their decision and outlining what is needed to prepare a revision will be listed below. The following reviewer(s) agreed to reveal their identity: Arvid Guterstam.

# Synthesis

This manuscript has undergone review by two experts in the field of gaze perception. Their detailed feedback is appended below for your consideration. Both reviewers have raised important points regarding further analyses, methodological clarifications, and the interpretation of your findings, which I strongly encourage you to address comprehensively.

A common thread in the reviews is the emphasis on substantiating the null findings presented in your study. I agree with reviewer 1 that null results are, in principle, scientifically valuable, and eNeuro is committed to publishing any type of result that is based on rigorous research. However, I also agree that such findings should ideally demonstrate that the null finding was not due to an ineffective experimental manipulation, confounds (e.g. due to eye movements), or insufficient data quality. In this vein, I concur with Reviewer 1's suggestion that including a positive control could significantly strengthen your manuscript by affirming that the observed null findings are not due to the reasons mentioned above.

Finally, I would like to ask you to consider making data and code publicly available.

# Reviewer 1

In this study, the authors compared BOLD activity within the gaze following patch (GFP) during gaze-direction following versus arrow-direction following. Contrary to their initial hypothesis, they did not find a significant difference between gaze and arrow-direction following, and were not able to reproduce the GFP as presented in previous studies. They specualte that a possible explanation for these negative results is that the gaze stimuli in the present study, in contrast to previous studies, did not contain an obvious change of direction that represented a visual motion.

This study addresses an important topic relating to the role of the GFP in gaze-following. The results are negative across the board; there is not a single activation or contrast that survive adequate correction for multiple comparisons. Interpreting negative results is notorously difficult, and I therefore commend the authors for submitting their negative results for publication instead of putting them in the drawer. That said, I have some major concerns/comments.

1. In the significance statement, the authors state: "This study presents evidence that the activation of a brain area previously thought to be a domain-specific cortical module specialized in the processing of other people's direction of visual attention does, in fact, depend on the presence of visual motion. Therefore, it must be characterized in a domain-general sense." This conclusion cannot be drawn from the presented data due to a lack of a positive control for the role of apparent motion (which the author acknowledge in the Discussion), and therefore the conclusions need to be toned down.

2. It would be very interesting to employ multivoxel pattern analysis (MVPA) to the current data set. MVPA is arguably more sensitive than simple univariate contrasts and relevant information regarding gaze-following in GFP might be contained in the fine-grained relative activities of neighbouring voxels, information that is lost in univariate contrasts. If gaze-following vs iris-color mapping can be decoded significantly better than chance from patterns of BOLD activity in the GFP, and if the GFP is domain-specific, one would predict that a classifier trained on gaze-following vs iris-color mapping cannot generalize to decode arrow-direction following versus arrow-color mapping. However, if the alternative hypothesis is true, and the GFP is domain-general, one would expect the classifier to generalize. Using this approach, it might be possible to directly contrast the two alternative hypotheses.

3. Since the results are negative and a positive control for the role of apparent motion is lacking, the conclusions of the paper in its current form are speculative. I leave it up to the editor to determine whether this work meets the standards for publication in eNeuro.

# Reviewer 2

Results:

page 8. "The HRFs for the mGFP show a statistically significant (95% CIs do not include the activation level of 0) deflection around 5 sec after cue onset in both hemispheres in all conditions but the iris-color condition". How does the author decide on the 5sec timepoint? What is the rationale/motivation on choosing this particular time? How long do we expect to see this effect last?

Methods:

Figure 1. "After cue presentation the participants had to wait with their response until the central fixation dot disappeared". How does the author make sure the participants look at central fixation during the process? If participants make saccades/eye movements before the central fixations, how did the author deal with the data?

For trials when the participants look at the correct/incorrect target, was there a difference in the results?

What eye tracker did the author use? What are the eyetracker specs? How did the author implement eyetracking calibration and set up?

Discussion:

The author proposed in discussion "As a third possibility, we propose that the observed activity patterns can be attributed to an effect described as implied motion. " I have trouble understanding that if the arrow-direction following case (without apparent motion) elicits siginificatn results in mGFP, why would be that the gaze-direction following does not show this "implied motion" effect? Could there be any difference in temporal development in these two cases? Maybe author could add more explanation in the discussion section.

## Author Response

# Synthesis This manuscript has undergone review by two experts in the field of gaze perception. Their detailed feedback is appended below for your consideration. Both reviewers have raised important points regarding further analyses, methodological clarifications, and the interpretation of your findings, which I strongly encourage you to address comprehensively. A common thread in the reviews is the emphasis on substantiating the null findings presented in your study. I agree with reviewer 1 that null results are, in principle, scientifically valuable, and eNeuro is committed to publishing any type of result that is based on rigorous research. However, I also agree that such findings should ideally demonstrate that the null finding was not due to an ineffective experimental manipulation, confounds (e.g. due to eye movements), or insufficient data quality. In this vein, I concur with Reviewer 1's suggestion that including a positive control could significantly strengthen your manuscript by affirming that the observed null findings are not due to the reasons mentioned above. Authors' answer: This point is indeed of utmost importance and we are aware that the lack of a positive control is the study's greatest weakness, limiting the interpretability of the first part of the analysis (BOLD-contrasts). Unfortunately, for organizational reasons (several of the subjects we tested are no longer available in Tübingen), it is not possible to add a meaningful positive control experiment post hoc. Such a control experiment would have to be based on a new group of subjects, therefore limiting its value. Hence, in view of the need to compare different populations of subjects we argue that adding such a control experiment would not strengthen the study more than referring to our old studies on the GFP including for example Marquardt et al., 2017, eNeuro, from which we took the stimuli with the modification described in the manuscript. (You may remove the latter sentence when handing over our answers to the reviewers but we also agree to open up the blinding if it helps to clarify this point.) That said, we do argue that the results presented in the study are not solely negative; even though we were not able to reproduce the GFP using the univariate methods used previously (Fig. 2, Tab. 1) we were able to demonstrate a statistically significant activation in the ROI analysis in all but the Iris-color mapping condition and importantly, that this BOLD activation is strongest in the 1 Arrow-direction following condition (Fig. 3). This result alone demonstrates that the GFP is not a dedicated gaze-module, as it was proposed in earlier studies. This is the main reason why we think the study is worth publishing without having the aforementioned positive control condition for the influence of visual motion. We thank you and the reviewers for having pointed out this issue and we have revised the manuscript to discuss this important consideration in more detail (pp. 10/11, 12 and 14). Finally, I would like to ask you to consider making data and code publicly available. We have added the URLs to the resources to the manuscript. # Reviewer 1 In this study, the authors compared BOLD activity within the gaze following patch (GFP) during gaze-direction following versus arrow-direction following. Contrary to their initial hypothesis, they did not find a significant difference between gaze and arrow-direction following, and were not able to reproduce the GFP as presented in previous studies. They speculate that a possible explanation for these negative results is that the gaze stimuli in the present study, in contrast to previous studies, did not contain an obvious change of direction that represented a visual motion. This study addresses an important topic relating to the role of the GFP in gaze-following. The results are negative across the board; there is not a single activation or contrast that survive adequate correction for multiple comparisons. Interpreting negative results is notoriously difficult, and I therefore commend the authors for submitting their negative results for publication instead of putting them in the drawer. That said, I have some major concerns/comments. 1. In the significance statement, the authors state: "This study presents evidence that the activation of a brain area previously thought to be a domain-specific cortical module specialized in the processing of other people's direction of visual attention does, in fact, depend on the presence of visual motion. Therefore, it must be characterized in a domain-general sense." This conclusion cannot be drawn from the 2 presented data due to a lack of a positive control for the role of apparent motion (which the author acknowledge in the Discussion), and therefore the conclusions need to be toned down. Authors' answer: We fully agree and we have revised the significance statement as follows: Significance Statement. This study presents evidence against the notion of domain-specificity of an area in the posterior temporal cortex (the gaze-following-patch; GFP) previously described to specifically serve eye gaze following. This conclusion is suggested by the finding that using arrows to identify a target object among distractors is accompanied by a comparable or even larger BOLD response than when the participants are asked to use the gaze direction of a demonstrator face for target selection. The fact that even the best candidate to date, the posterior temporal GFP, does not stand up to critical scrutiny casts doubt on the assumption that the brain uses a specific module to enable gaze following, as proposed by Simon Baron-Cohen. 2. It would be very interesting to employ multivoxel pattern analysis (MVPA) to the current data set. MVPA is arguably more sensitive than simple univariate contrasts and relevant information regarding gaze-following in GFP might be contained in the fine-grained relative activities of neighboring voxels, information that is lost in univariate contrasts. If gaze-following vs iris-color mapping can be decoded significantly better than chance from patterns of BOLD activity in the GFP, and if the GFP is domain-specific, one would predict that a classifier trained on gaze-following vs iris-color mapping cannot generalize to decode arrow-direction following versus arrow-color mapping. However, if the alternative hypothesis is true, and the GFP is domain-general, one would expect the classifier to generalize. Using this approach, it might be possible to directly contrast the two alternative hypotheses. Authors' answer: We have considered using MVPA and especially cross-condition decoding. However, we came to the conclusion that this would not help to resolve the alternative of domain-specifity vs. generality for the following reason: We think that it is false to assume that if we cannot decode arrow-direction vs arrow-color using a decoder trained on gaze-direction vs iris-color, then the GFP could be considered to be domain specific. This is because if the GFP were a "domain-general early visual" area its activity would 3 depend on the low-level features of the stimuli, which are very different between the two (face and arrows). This alone would hinder successful cross-decoding without the necessity of domain-specificity. On the other hand, if we could decode we would not have learned more than we already have from the univariate analysis and the hrf modeling. The only interesting result here would be the inability to decode arrow-direction vs arrow-color at all (meaning also not when trained on arrow-direction vs arrow-color) but only gaze-direction vs iris-color. However this appears unlikely given that the activation during both arrow conditions is actually stronger - but differential - than during the gaze/iris conditions (see Fig. 3). If the reviewer thinks and convinces us that this argument is erroneous, we would be happy to reconsider applying MVPA. 3. Since the results are negative and a positive control for the role of apparent motion is lacking, the conclusions of the paper in its current form are speculative. I leave it up to the editor to determine whether this work meets the standards for publication in eNeuro. Authors' answer: We fully agree with this reviewer´s criticism regarding the limited interpretation concerning the role of visual motion given the lack of a positive control. Nevertheless we think that the second part of the analysis, the HRF estimations, does provide direct evidence that the GFP does not differentiate between an arrow pointing towards an object and a face gazing at an object irrespective of the role of visual motion. We have toned down the discussion regarding the statements on the role of visual motion and emphasized the ROI analysis more. (see also our answer to the Synthesis) # Reviewer 2 Results: page 8. "The HRFs for the mGFP show a statistically significant (95% CIs do not include the activation level of 0) deflection around 5 sec after cue onset in both hemispheres in all conditions but the iris-color condition". How does the author decide on the 5sec timepoint? What is the rationale/motivation on choosing this particular time? How long do we expect to see this effect last? 4 Authors' answer: Here, we have assumed - without justification - that the reader is familiar with the standard hemodynamic response function. Pointing out the 5 second time point reflects the peak of the canonical HRF, i.e. it is not a time point that we chose but reflects the canonical maximum of the BOLD response. We have clarified this in the manuscript. Methods: Figure 1. "After cue presentation the participants had to wait with their response until the central fixation dot disappeared". How does the author make sure the participants look at central fixation during the process? If participants make saccades/eye movements before the central fixations, how did the author deal with the data? For trials when the participants look at the correct/incorrect target, was there a difference in the results? What eye tracker did the author use? What are the eyetracker specs? How did the author implement eyetracking calibration and set up? Authors' answer: This is a valid criticism. We have recorded eye-tracking data during the fMRI recordings. However, the recordings suffered from bad quality and could not be analyzed consistently. When we tried, we were able to analyze only ~30% of trials. In ~10% of these "good" trials, participants made an error. This was the case for all experimental conditions and roughly matches the behavioral results reported in Marquardt et al. 2017, eNeuro. Further, the eye-tracking camera enabled us to manually monitor participants' behavior during the experiment, giving us confidence that participants generally complied with the task requirements. Before the fMRI session participants underwent a practice session in which their behavior was closely monitored, and corrected, as well. This, together with the fact that the task was easy and intuitive makes us confident that the number of error trials was indeed small and, thus, can be ignored. We have added this reasoning to the manuscript. That said, it would indeed be super interesting to specifically analyze error trials. However this would require a different experimental design and especially more successful efforts in recording eye-tracking data. Discussion: The author proposed in discussion "As a third possibility, we propose that the observed activity patterns can be attributed to an effect described as implied 5 motion. " I have trouble understanding that if the arrow-direction following case (without apparent motion) elicits significant results in mGFP, why would be that the gaze-direction following does not show this "implied motion" effect? Could there be any difference in temporal development in these two cases? Maybe author could add more explanation in the discussion section. Authors' answer: We think there is a misunderstanding here (we have revised the respective paragraph in the manuscript). There is indeed a significant activation in the gaze-following condition, as well! However, the response correlated with gaze-following appears to be smaller than the one related to arrow-direction following (see Fig. 3). We find this result particularly interesting because it is at odds with a finding from Michael Graziano's lab, who reported that there is no "implied motion effect" when observing arrows (see Discussion).
